# Deregulated MicroRNAs in Myotonic Dystrophy Type 2

**DOI:** 10.1371/journal.pone.0039732

**Published:** 2012-06-29

**Authors:** Simona Greco, Alessandra Perfetti, Pasquale Fasanaro, Rosanna Cardani, Maurizio C. Capogrossi, Giovanni Meola, Fabio Martelli

**Affiliations:** 1 IRCCS-Policlinico San Donato, Milan, Italy; 2 University of Milan, Milan, Italy; 3 Istituto Dermopatico dell’Immacolata-IRCCS, Rome, Italy; University of Rome La Sapienza, Italy

## Abstract

Myotonic Dystrophy Type-2 (DM2) is an autosomal dominant disease caused by the expansion of a CCTG tetraplet repeat. It is a multisystemic disorder, affecting skeletal muscles, the heart, the eye, the central nervous system and the endocrine system. Since microRNA (miRNA) expression is disrupted in Myotonic Dystrophy Type-1 and many other myopathies, miRNAs deregulation was studied in skeletal muscle biopsies of 13 DM2 patients and 13 controls. Eleven miRNAs were deregulated: 9 displayed higher levels compared to controls (miR-34a-5p, miR-34b-3p, miR-34c-5p, miR-146b-5p, miR-208a, miR-221-3p and miR-381), while 4 were decreased (miR-125b-5p, miR-193a-3p, miR-193b-3p and miR-378a-3p). To explore the relevance of DM2 miRNA deregulation, the predicted interactions between miRNA and mRNA were investigated. Global gene expression was analyzed in DM2 and controls and bioinformatic analysis identified more than 1,000 miRNA/mRNA interactions. Pathway and function analysis highlighted the involvement of the miRNA-deregulated mRNAs in multiple aspects of DM2 pathophysiology. In conclusion, the observed miRNA dysregulations may contribute to DM2 pathogenetic mechanisms.

## Introduction

Myotonic Dystrophy (DM) is an autosomal dominant disease and the most common muscular dystrophy of the adult, with a prevalence of 1/8,000 worldwide [1]. DM is a progressive multisystemic disorder characterized by a variety of manifestations that include muscle hyperexcitability (myotonia), muscular dystrophy, cardiac conduction defects, dilated cardiomyopathy, insulin-resistance, cataracts, neurological involvement, and a multiplicity of serological abnormalities. The severity and the number of symptoms are highly variable and can range from minimal signs, to facial and muscle wasting and weakness, to severe congenital disorder [1]. Respiratory failure and cardiac arrhythmias are among the main causes of premature death in DM patients [1]. At the histological level, pathological features of the DM skeletal muscle include the concomitant presence of atrophic and hypertrophic myofibers, increased number of central nuclei, and presence of fibers with nuclear clumps [2].

Two types of DM have been described. The most common form of DM, named type 1 or Steinert’s disease (DM1, OMIM 160900), is caused by an expanded (CTG)n in the 3′ untranslated region of the *Dystrophia Myotonica Protein Kinase* gene [3], [4], [5]. The second form of DM is named type 2 or PROximal Myotonic Myopathy-PROMM (DM2, OMIM 602688): it displays a prevalently proximal impairment and milder clinical symptoms than DM1, since it does not show a congenital form or anticipation i.e. a phenomenon of progressively earlier and more severe manifestation of the disease. DM2 is caused by the expansion of a tetranucleotidic repetition (CCTG)n in the first intron of the CCHC-type zinc finger, nucleic acid binding protein (CNBP) [6].

Several lines of evidence indicate that an RNA-mediated toxic gain-of-function is the common pathogenetic mechanism of the two DM types. In both DM1 and DM2, the mutation causes the accumulation of the expanded CUG/CCUG transcripts into discrete nuclear RNA foci. The expanded RNA that is trapped in the nucleus, deregulates the interaction of RNA binding proteins, which are essential for mRNA splicing and other cell processes, leading to aberrant RNA maturation and cell dysfunction [7].

MicroRNAs (miRNAs) are short non-coding RNAs that regulate the stability and/or the translational efficiency of target mRNAs. miRNAs have a very pervasive role: bioinformatic studies estimate that >60% of all transcripts are regulated by one or more miRNA [8]. Indeed, miRNAs have a demonstrated impact on virtually all cell functions, including differentiation, proliferation and apoptosis [9], [10]. Specifically, miRNA sequences can regulate skeletal and cardiac muscle function in both development and disease, such as ischemia and muscular dystrophy [11], [12].

We and others demonstrated miRNA deregulation in Duchenne Muscular Dystrophy patients and in *mdx* mice, both lacking a functional *dystrophin* gene [13], [14], [15], [16]. Moreover, Eisenberg and collaborators described miRNA expression profiles in skeletal muscle from various human primary muscle disorders and identified a series of miRNAs that are deregulated in almost all myopathies analyzed [14]. Finally, we and others analyzed the expression of miRNAs in DM1 and identified specific miRNAs displaying altered expression and localization [17], [18], [19].

In this study, miRNA expression profiles were measured in the skeletal muscle of DM2 patients. We identified a subset of miRNAs that are specifically deregulated potentially contributing to DM2 pathogenetic mechanisms.

**Table 1:** Characteristics of Profiling Cohorts (Mean±Se).

PARAMETER	DM2 (N = 13)	CONTROLS (N = 13)	p
**AGE (years)**	56.9±1.9	49.3±2.5	ns
**SEX (f/m)**	5/8	5/8	ns
**STRENGHT (biopsied muscle) (MRC scale)**	4.5±0.1	4.9±0.04	ns
**STRENGHT (Megascore on 15 muscles, MRC scale)**	137.4±9.1	145.6±6.8	ns
**MYOTONIA (%)**	85	0	<0.0001
**GLUCOSE (mg/dl) (n.v.70–110)**	101.4±16.0	86.0±8.5	ns
**DIABETES MELLITUS (%)**	18	0	ns
**CPK (IU/l) (n.v. m<190, f<167)**	292.7±3.5	236.4±67.1	ns
**FT3 (pg/ml) (n.v. 1.5–4.1)**	3.0±3.1	1.7±0.5	ns
**FT4 (ng/dl) (n.v. 0.8–1.9)**	1.3±0.3	1.2±0.3	ns
**TSH (mIU/ml) (n.v. 0.4–4.0)**	1.0±0.2	0.7±0.2	ns
**ECG-QRS duration (msec) (n.v. 60–100)**	94.3±16.4	85.9±12.1	ns
**EF (%) (n.v.>50)**	54.5±9.7	64.0±19.3	ns
**ICM (%)**	9	0	ns
**SERIC β-GLOBULIN (%)**	12.3±1.7	14.1±2.0	ns
**CATARACT (%)**	70	0	<0.0005

**Abbreviations: MRC scale** = Medical Research Council scale (0–5 grade); **CPK** = Creatine PhosphoKinase; **FT3** = free-triiodothyronine; **FT4** = free-thyroxine; **TSH** = thyroid stimulating hormone; **ECG** =  electrocardiogram; **QRS duration** = QRS complex of ECG corresponds to the depolarization of the right and left ventricles of the human heart; **EF** = ejection fraction; **ICM** = ischemic cardiomyopathy.

## Materials and Methods

### Patient Selection and Skeletal Muscle Biopsies

This study was authorized by the Institutional Ethics Committee (ASL MI2) and was conducted according to the principles expressed in the Declaration of Helsinki, the institutional regulation and Italian laws and guidelines. All biopsy specimens were taken after specific written informed consent was obtained. Human muscle biopsies from biceps brachii were harvested under sterile conditions and snap-frozen in liquid nitrogen. Clinical diagnosis of DM2 patients was based upon the criteria set by the International Consortium for Myotonic Dystrophies [20]. Fluorescence *in situ* hybridization was performed on frozen muscle sections to confirm DM2 diagnosis according to Cardani et al. [21]. Control (CTR) biopsies were from subjects admitted with suspected neuromuscular disorder of undetermined nature. CTR biopsies did not show overt signs of muscle pathology on histological and immunohistochemical examination. All muscle biopsies were processed by the same pathology team. Immunohistochemical analysis of atrophy and hypertrophy in fast and slow fibers was carried out as previously described [22].

### RNA Isolation and miRNA Profiling

Total RNA was extracted by Trizol (Invitrogen) and the TissueLyser system (Qiagen). TaqMan Low Density miRNA arrays v.2 (Applied Biosystems) were used to measure 365 human mature miRNAs, as previously described [23]. After median Ct value normalization, relative miRNA expression was calculated with the 2^−ΔΔCt^ method [24]. We further analyzed those miRNAs with a Ct≤33, displaying a significant fold change ≥2. Data distribution was evaluated by the Kolmogorov-Smirnov’s normality test. Permutational multivariate analysis of variance was performed by the multivariate non-parametric PERMANOVA test, using 9,999 permutations, Bonferroni post-hoc test and Gower test for distance measurement. Adjusted p<0.05 was considered as statistically significant.

### mRNA Profiling

Gene expression profiles were measured using the Gene Chip Human Exon 1.0 ST Array (Affymetrix). One hundred ng of total RNA were analyzed according to the manufacturer instructions. Data analysis was computed using Partek Genomics Suite (Partek Inc.) according to MIAME guidelines. The output signals were normalized using the Partek Robust Multi-Chip Average analysis adjusted for the GC content of probe sequence (GCRMA). The genes that were differentially expressed among the two classes were identified using the Analysis of Variance (ANOVA) as implemented in the Partek Gene Expression tool. An estimate of the associated False Discovery Rate (FDR) was computed per gene using the method of Benjamini and Hochberg [25]. mRNA transcripts were considered to be significantly differentially expressed if they obtained ANOVA p-value<0.005 and FDR<0.15.

### qPCR Assay

Individual mature miRNAs were measured using TaqMan MicroRNA single assays (Applied Biosystems) and samples were normalized to miR-16 expression [26]. MiRNA profiling indicated that miR-16 was not-modulated in DM2 patients. mRNA levels were measured using the SYBR-GREEN qPCR method (Applied Biosystems) and samples were normalized to GAPDH. For both miRNAs and mRNAs, relative expression was calculated using the comparative Ct method 2^–ΔΔCt^ [24]. Each sample was measured in triplicate and values were averaged.

### DM2 miRNA Score

To calculate the DM2 miRNA scores, the median fold change of all the deregulated miRNAs was calculated for each subject. For negatively regulated miRNAs, the minus sign was inverted before calculating the median.

### Bioinformatic Analysis

Bioinformatic prediction of miRNA target genes was performed using miRonTop [27]. Predicted miRNA target genes were compared to differently expressed genes (p≤0.01) displaying an opposite modulation with their corresponding miRNA. The correlation analysis between mRNA and miRNA expression was evaluated by the quantitative trait tool in BRB ArrayTools (Richard Simon and BRB-ArrayTools Development Team) (p≤0.01).

Pathway analysis was performed using Ingenuity Pathways Knowledge Base (version 8.8, Ingenuity Systems) as reference set and assuming direct and indirect relationships. A Fisher’s exact test p-value <0.05 was deemed as statistically significant.

### Statistics

Continuous variables are expressed as mean±standard error (SE), unless indicated differently. For group-wise comparisons, Mann–Whitney test or *t*-test were used as appropriate. For the DM2 miRNAs-score, Mann-Whitney’s variance analysis was used. The ability to discriminate between the DM2 and control groups was characterized by the receiver operating characteristic (ROC) curve, and the area under the ROC curve (AUC) was calculated. Spearman rank correlation was used to compare the DM2 miRNA score with atrophy/hypertrophy fiber index. All tests were performed 2-sided and a p≤0.05 was considered as statistically significant. For statistical analysis and heat map data visualization, GraphPad Prism v.4.03 (GraphPad Software Inc.) and Genesis 1.7.2 (Graz University of Technology, Institute for Genomics and Bioinformatics) softwares were used, respectively.

## Results

### Identification of Differentially Expressed miRNAs in DM2 Patients

**Figure 1:**
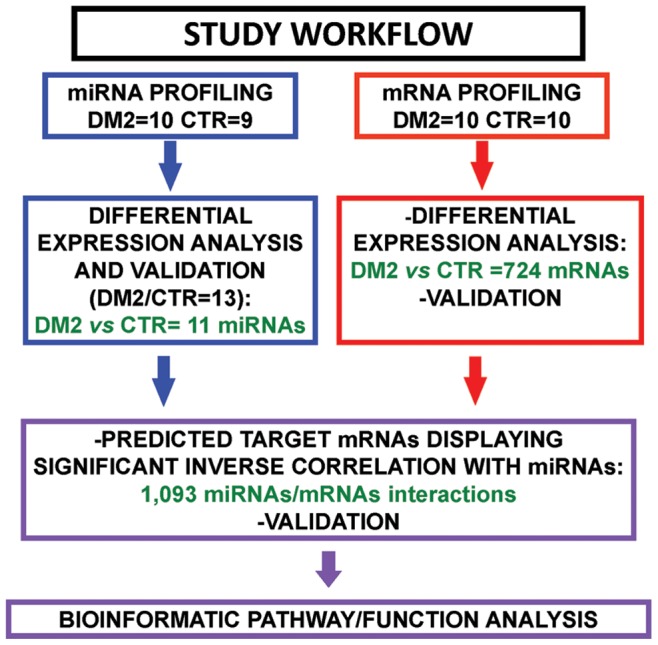
Experimental Plan. miRNA (blue) and mRNA (red) expression patterns were determined in DM2 and CTR, and significantly modulated miRNAs and mRNAs were identified. Validation was performed by qPCR for both miRNAs and mRNAs in 13 DM2 patients and 13 CTR. miRNA targets among the mRNAs identified by Class Comparison analysis were predicted by dedicated softwares. Only miRNA/mRNA couples displaying inverse significant correlation were selected and then analyzed by Ingenuity Pathway Analysis software that allowed to identify enriched molecular pathways and functions.

Patients were selected so that DM2 and controls (CTR), displayed similar age and sex distribution (Table 1). Most DM2 patients had myotonia and cataract, two disease hallmarks, while differences in other clinical parameters were not significant. The experimental plan is schematized in Figure 1.

**Figure 2:**
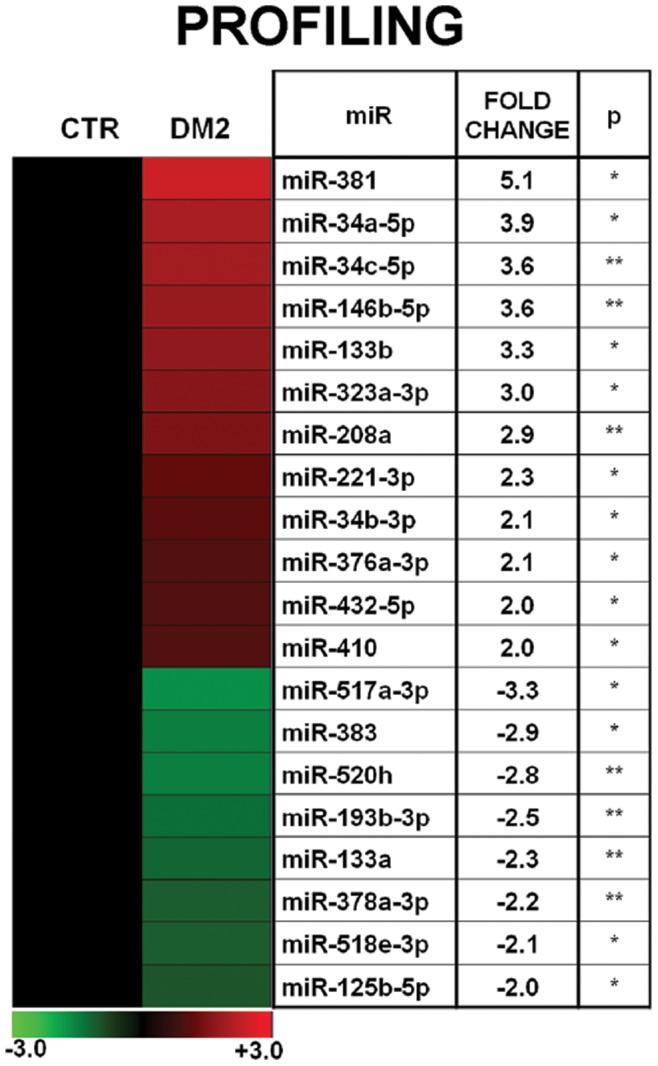
Profiling of miRNAs in DM2 patients and CTR. In the heat map on the left, mean miRNAs expression values are shown in a log_2_ scale (-ΔΔCt), where red and green indicate positive and negative modulation respectively. The table on the right shows the same values in a linear scale (DM2 = 13, CTR  = 13; *p≤0.05 **p≤0.01).

**Figure 3:**
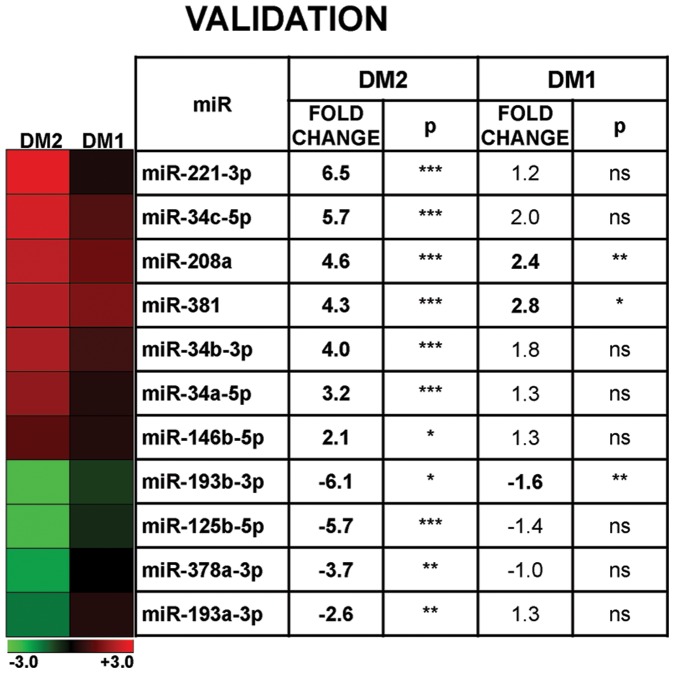
Validation of miRNA modulations in DM1 and DM2 patients. In the heat map on the left, the mean values of miRNA expression in both DM1 and DM2 compared to controls are shown in a log_2_ scale (-ΔΔCt), where red and green indicate positive and negative modulation respectively. On the right, the table shows the same values in a linear scale; statistically significant differences are highlighted in bold (DM2 n = 13, DM1 = 16, CTR n = 13; *p≤0.05 **p≤0.01 ***p≤0.001).

Skeletal muscle biopsies were harvested from 10 DM2 patients and 9 CTR, and total RNA was extracted. MiRNA profiling showed that, out of 365 miRNA measured, 203 and 199 were readily detectable (Ct<33) in DM2 and CTR groups, respectively (see Gene Expression Omnibus database, GEO, GSE37794 for a complete gene list). Statistical analysis showed that 20 miRNAs were significantly modulated ≥2-fold in DM2 compared to CTR (Fig. 2) and these miRNAs were validated by more sensitive and specific qPCR assays. This validation step included the same patients and CTR used for profiling, and was extended to three more DM2 and four more CTR (n = 13 for each group). Moreover, we added six more miRNAs to the 20 modulated miRNAs: the muscle-specific miRNAs, miR-1 and miR-206 [28], [29], miR-221-3p, which is co-transcribed with miR-222-3p, miR-193a-3p and miR-146a since they belong to miR-193 and miR-146 families, respectively, and miR-499-5p, which is downstream of miR-208a network [30]. We found that 11 miRNAs were validated as significantly deregulated in DM2 patients (Fig. 3).

These DM2 miRNAs were also tested in an age and sex matched cohort of DM1 patients. We found that miR-193b-3p, miR-208a and miR-381 showed a similar significant modulation also in these patients (Fig. 3).

### A DM2 miRNA Score Discriminates between Patients and Controls and Correlates with Myofiber Atrophy and Hypertrophy

**Figure 4:**
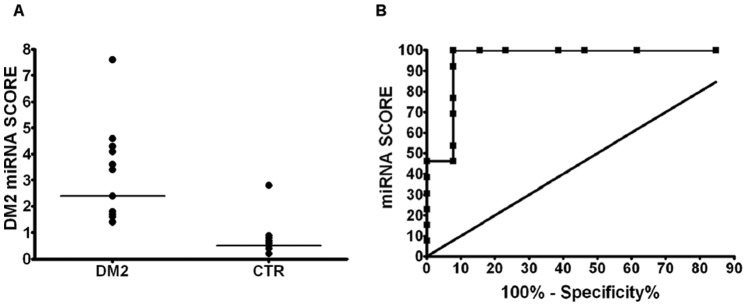
Discrimination between DM2 and control groups using the DM2 miRNA score. (A) Dot plot of the DM2 miRNA score in DM2 patients and CTR. The segments among the dots indicate median values for each group and the difference is statistically significant (DM2 = 13, CTR n = 13; p<0.0001). (B) ROC curve displaying DM2 miRNA score discrimination between the DM2 and CTR groups (AUC = 0.96).

DM2 deregulated miRNAs were also considered collectively: median fold change of the differentially expressed miRNAs was used to calculate a “DM2 miRNAs score” in each patient or CTR subject. The DM2 miRNA score allowed a significant separation between the DM2 and the CTR groups, with a median score of 2.3 in the DM2 group and 0.6 in the CTR group (Figure 4A). The ability of the DM2 miRNA score to discriminate DM2 patients from CTR was also demonstrated by the Receiver-Operator Characteristic (ROC) curve (Figure 4B). A ROC curve allows to visualize the reciprocal relationship between sensitivity and specificity when discriminating between control and DM2 values. The calculation of sensitivity and specificity depends on the threshold value used to separate the two classes; if the threshold value used is high, the specificity of the test increases, but the sensitivity decreases, conversely if the threshold is low, the test’s sensitivity is increased [31], [32]. By using a threshold score of 1.15 we achieved a sensitivity of 100% and a specificity of 92.3% for the identification of DM2 patients. Moreover, the Area Under Curve (AUC), which quantifies the overall ability of the test to discriminate between normal and diseased individuals, was 0.96, where perfect test (with zero false positives and zero false negatives) has an area of 1.00 [33].

**Figure 5:**
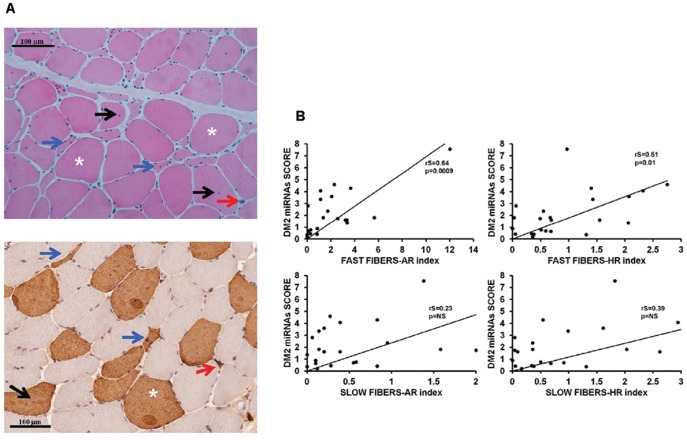
Correlation of atrophy and hypertrophy with DM2 miRNAs score. A) Representative stainings of transverse sections of frozen DM2 muscle biopsies. Top panel shows a hematoxylin and eosin staining; bottom panel shows an immunostaining for fast myosin heavy chain. Atrophic (*blue arrows*) and hypertrophic (*asterisks*) myofibers, as well as nuclear clumps (*red arrows*) and internal nuclei (*black arrows*) are highlighted. B) Spearman’s correlation between DM2 miRNAs score and Atrophy (AR) or Hypertrophy (HR) indexes in fast and slow fibers (DM2 n = 13; CTR n = 9).

At the histological level, a DM2 hallmark is represented by the concomitant presence of atrophic and hypertrophic myofibers, preferentially in type 2 (fast) fibers [2], [34], [35] (Figure 5A). We found that DM2 miRNA score correlated with both the atrophy (AR) and the hypertrophy (HR) index, but only in type 2 myofibers (Figure 5B).

### Transcriptome Analysis and Predicted miRNA/mRNA Interactions

To investigate the implications of miRNA dysregulations identified in DM2 patients, we tested their impact on the transcriptome using high-density oligonucleotide microarrays. The same RNAs used for miRNA profiling were analyzed, with the addition of one extra control (total, 10 DM2 and 10 CTR) (see Gene Expression Omnibus database, GEO, GSE37084 for a complete gene list). As expected, unsupervised hierarchical clustering analysis segregated the profiles of DM2 and CTR (Fig. S1). Class Comparison analysis versus the CTR group was performed identifying 724 non redundant transcripts differentially expressed in DM2 patients. qPCR validation allowed to confirm 10 out of 12 tested genes (not shown).

Next, we attempted to identify the contribution of miRNA deregulation to differential gene expression. Given the inhibitory nature of miRNAs [8], [36], mRNA displaying reciprocal modulation to DM2 miRNAs were selected. These transcripts were further filtered using target identification algorithms, to predict direct binding events. The application of these three sequential filers allowed the identification of 1,093 miRNA/mRNA interactions (Table S1).

**Figure 6:**
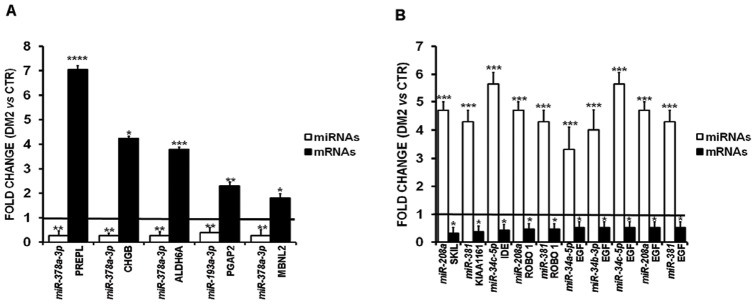
Validation of miRNA/mRNA interactions in DM2 patients. Validation by qPCR of the expression of randomly selected mRNAs either up-(**A**) and down-(**B**) regulated in DM2 patients vs CTR. Expression level compared to CTR of the targeting miRNA is indicated for each mRNA (DM2 = 12, CTR = 10;*p≤0.05; **p≤0.01; ***p≤0.001; ****p≤0.0001).

It is worth noting that several mRNAs were predicted targets of more than one miRNA deregulated in DM2, with a potential signal reinforcement. A randomly selected subset of the identified miRNA-target modulations was further tested by qPCR and 15 out of 22 were validated (Fig. 6). The remaining 7 interactions originated from 3 transcripts (CARM1, NXN and YIPF7) that were not detectable in most controls and DM2 samples.

### Pathways Analysis of Gene Expression Changes

To facilitate the interpretation of the complex gene expression changes observed, we used Ingenuity Pathway Analysis software (IPA) exploring enriched biological functions and pathways. Both global gene expression changes and DM2 miRNA/mRNA interactions were analyzed.

To assess the potential functional relevance of the overall gene expression changes observed in DM2 patients, differentially expressed genes, analyzed by Class Comparison, were uploaded to the IPA database. Several interesting pathways and functions were enriched significantly in the DM2 group compared to CTR, such as “Mitochondrial Dysfunctions”, “TGF-β Signaling”, “Insulin Receptor Signaling”, “Calcium Signaling” for the pathways and “Skeletal and Muscular System Development”, “Cell Cycle”, “Cell Death”, “Skeletal and Muscular Disorders” for the enriched functions (Table S2 and S3). Moreover, “Cardiac Dilation”, “Cardiac Hypoplasia” and “Cardiac Artheriopathy” among the toxic functions were also found enriched (Table S4). Complete lists of enriched categories can be found in Table S8.

IPA analysis of miRNAs predicted targets differentially modulated in DM2 patients identified several important pathways enriched, such as: “Insulin Receptor Signaling”, “Integrin Signaling”, “PI3K/AKT Signaling” and “TGF-β Signaling” (Table 2 and Table S5). “Skeletal and Muscular Disorders”, “Ophthalmic Diseases” and “Endocrine System Disorders” and “Cardiac Damage”, “Cardiac Dilation” and “Heart Failure” were relevant enriched functions (Table 2 and Table S6) and toxic functions (Table 2 and Table S7), respectively, and all the enriched categories are showed in Table S9, where the selected categories of Table 2 are highlighted in bold.

**Table 2:** Pathways and Functions Analysis by IPA.

PATHWAYS	p-value	ratio	enrichment index
**Insulin Receptor Signaling**	0.002	11/139	263
**Integrin Signaling**	0.003	14/209	223
**FAK Signaling**	0.02	7/102	345
**PI3K/AKT Signaling**	0.03	8/140	285
**RAR Activation**	0.03	10/183	275
**TGF-β Signaling**	0.04	6/89	478
**FUNCTIONS**	**p-value**	**ratio**	**enrichment index**
**Skeletal and Muscular System Development and Function**	7.8E−06	50/2849	15
**Skeletal and Muscular Disorders**	2.7E−04	142/3851	11
**Neurological Disease**	2.4E−04	179/4743	9
**Cardiovascular Disease**	9.4E−04	110/2663	159
**Metabolic Disease**	9.4E−04	124/2496	17
**Endocrine System Disorders**	1.4E−03	10/2271	19
**Cell Growth and Proliferation**	2.1E−03	36/2884	15
**Cardiovascular System Development and Function**	2.7E−03	11/2738	15
**Ophthalmic Disease**	2.7E−03	3/987	43
**TOXIC FUNCTIONS**	**p-value**	**ratio**	**enrichment index**
**Cardiac Damage**	3.0E−02	1/88	481
**Cardiac Dilation**	3.0E−02	4/83	510
**Heart Failure**	4.8E−02	11/330	128

**Table legend:**

**ratio** = number of modulated genes/total number of genes present in the relevant IPA category.

**enrichment index** =  ratio between observed genes and number of genes expected by chance in each category.

## Discussion

Understanding the molecular mechanisms triggered by the tetranucleotide expansion in the *Zinc Finger Protein-9* gene is of great importance to gain insight into the molecular mechanisms underpinning DM2 disease. For the first time, we measured the miRNA profile of skeletal muscle biopsies of DM2 patients, identifying 11 deregulated miRNAs. We and others found that miR-221-3p, miR-381, miR-34a-5p, miR-34c-5p, miR-146b-5p, and miR-193b-3p were similarly modulated in muscular diseases other than DM2 [13], [14] (Table S10), suggesting potential similarities in certain aspects of the pathogenetic mechanisms. The DM2 miRNAs were also measured in DM1 patients and miR-193b-3p, miR-208a and miR-381 were similarly modulated. The differences displayed by the remaining miRNAs did not reach statistical significance, possibly due to insufficient patient numerosity.

We found that a DM2 miRNA score allowed to discriminate DM2 patients from CTR with a good sensitivity and specificity. Furthermore, DM2 miRNA score correlated to atrophy and hypertrophy of type 2, fibers that are particularly affected in DM2, indicating that the deregulated miRNAs were related to the disease pathogenetic mechanism. Further studies are granted to explore DM2 miRNA score diagnostic potential.

The up-regulation of all members of miR-34 family appears particularly significant, given miR-34 role in cell differentiation, apoptosis and senescence [37]. It is also worth noting the up-regulation of miR-221-3p, playing an important role in myotube maturation and in the maintenance of the myofibrillar organization [38]. Finally, we demonstrated the down-regulation of miR-125b-5p, which negatively regulates myoblast differentiation and muscle regeneration [39].

In an attempt to gain further insight into the underlying molecular mechanisms, we investigated the transcripts predicted to be regulated by the DM2 miRNAs. In most circumstances, miRNA binding induces the degradation of the target mRNA [8], [40]. Thus, we measured the transcriptome of DM2 patients and used target prediction softwares [27] to identify the genes potentially regulated by the DM2 miRNAs, directly. To minimize the number of false positive miRNA/mRNA interactions, we used three selection criteria: 1) only significantly modulated miRNAs and mRNAs were considered; 2) given the inhibitory nature of miRNA function [8], [36], only miRNA/mRNAs couples displaying a statistically significant reciprocal correlation of their expression levels were included; 3) among the genes passing filters 1 and 2, predicted targets were identified by MiRonTop [27]. After this highly selective selection, 1,093 miRNA/mRNA interactions were identified. Several mRNAs were targeted by more than one miRNA, leading to a potential reinforcement of the final deregulation. For instance, EGF was a predicted target not only of the three members of the miR-34 family, but also of miR-208a and miR-381.

The increase of MBNL2, a miR-378a-3p predicted target, was another interesting finding. Indeed, a hallmark of DM2 is the nuclear accumulation of (CCUG)n repeats that bind and sequester muscleblind-like proteins MBNL1, MBNL2 and MBNL3 [41], [42], [43]. The observed induction of MBNL2 mRNA expression in DM2 may be an adaptive response counteracting the decreased bioavailability of MBNL.

To the best of our knowledge, this represents the first study investigating miRNA/transcriptome interactions in DM2 patients, paving the way to further investigations that are necessary to formally demonstrate cause/effect relationships between the DM2 miRNAs and their targets. Moreover, a potential caveat of our analysis is represented by the heterogeneity of the cell populations composing skeletal muscle tissue. Albeit myofibers constitute the majority of the tissue mass, it is possible that certain miRNAs and target mRNAs are deregulated in different cell types.

A bioinformatic analysis of the pathways and functions revealed that DM2 miRNA targets were potentially involved in most DM2 pathogenetic mechanisms. The functions “Skeletal and Muscular System Development and Function”, “Neurological Disease” and “Skeletal and Muscular Disorders” are of obvious relevance. The “PI3K/AKT signaling “and the “TGF-β signaling” pathways seem particularly important. Indeed, dysregulation of TGF-β signaling has been implicated in various pathological conditions affecting skeletal muscle, both inherited and acquired [44], and Akt2 expression is greatly increased during skeletal muscle cell differentiation and myocyte growth, suggesting a critical role of Akt2 in myogenesis [45], [46], [47]. Thus, both PI3K/AKT and TGF-β signaling may relate to the atrophic/hypertrophic myofiber alterations characterizing DM2.

DM2 is a multisystemic disease. Indeed, other pathways and functions are linked to aspects of DM2 that do not affect the skeletal muscle directly. The “Insulin Receptor Signaling” pathway as well as the “Metabolic Disease” and “Endocrine System Disorders” functions suggest the involvement of the relevant miRNA/mRNA couples in insulin resistance, a classic DM clinical feature, predisposing to Type 2 diabetes [1], [34], [48].

Albeit the assayed tissue was skeletal muscle, many functions and toxic functions related to heart and cardiovascular disease, in keeping with the cardiac conduction defects and the dilated cardiomyopathy commonly observed in DM. Another DM hallmark is cataract; thus, it was interesting that the “Ophthalmic Disease” function was also represented.

Looking at the global alterations of the transcriptome, to the best of our knowledge, only one previous study exists [22]. However, a direct comparison with our investigation is limited by the fact that Vihola et al. narrowed their scope to 327 probesets functionally associated with muscle *a priori*. Nevertheless, it is interesting to note that both studies identified several altered genes involved in Calcium handling and signaling, that may relate to the abnormal calcium handling and myotonia observed in DM [49], [50], [51]. Moreover, in keeping with increased oxidative stress levels measured in DM1 patients [52], we found that “NRF2-mediated Ox-Stress Response” and “mitochondrial dysfunction” pathways were also enriched.

### Conclusions

In conclusion, we identified a small subset of miRNA whose expression was deregulated in DM2. These findings may improve our understanding of the molecular mechanisms linking (CCTG)n expansion to disease.

## Supporting Information

Figure S1 **Unsupervised hierarchical clustering of mRNA expression differentiating DM2 (n = 10) from controls (n = 10).** Each row represents an mRNA, and each column represents an individual. A color code represents the relative intensity of the expression signal, with red indicating high expression and blue indicating low expression.
(PPT)

Table S1 **Transcriptome analysis identified 1,093 miRNA/mRNA interactions.** Table represents the identified miRNAs/mRNA interactions, where green and red colours indicate down- or up-regulation of miRNA or mRNA compared to controls, respectively.
(XLS)

Table S2 **Ingenuity Pathway Analysis (IPA) of important pathways, functions and toxic functions enriched in transcriptome.** Some important enriched pathways, functions and toxic functions and relative miRNA/mRNA interactions are shown; green and red colours indicate down- or up-regulation of miRNA or mRNA, respectively. Complete lists of enriched categories can be found in Table S8.
(XLS)

Table S3 **Ingenuity Pathway Analysis (IPA) of important pathways, functions and toxic functions enriched in transcriptome.** Some important enriched pathways, functions and toxic functions and relative miRNA/mRNA interactions are shown; green and red colours indicate down- or up-regulation of miRNA or mRNA, respectively. Complete lists of enriched categories can be found in Table S8.
(XLS)

Table S4 **Ingenuity Pathway Analysis (IPA) of important pathways, functions and toxic functions enriched in transcriptome.** Some important enriched pathways, functions and toxic functions and relative miRNA/mRNA interactions are shown; green and red colours indicate down- or up-regulation of miRNA or mRNA, respectively. Complete lists of enriched categories can be found in Table S8.
(XLS)

Table S5 **Ingenuity Pathway Analysis (IPA) of important pathways, functions and toxic functions enriched in miRNAs targets analysis.** Some important enriched pathways, functions and toxic functions in miRNA/mRNA interactions are shown; green and red colours indicate down- or up-regulation of miRNA or mRNA, respectively. Complete lists of enriched categories can be found in Table S9.
(XLS)

Table S6 **Ingenuity Pathway Analysis (IPA) of important pathways, functions and toxic functions enriched in miRNAs targets analysis.** Some important enriched pathways, functions and toxic functions in miRNA/mRNA interactions are shown; green and red colours indicate down- or up-regulation of miRNA or mRNA, respectively. Complete lists of enriched categories can be found in Table S9.
(XLS)

Table S7 **Ingenuity Pathway Analysis (IPA) of important pathways, functions and toxic functions enriched in miRNAs targets analysis.** Some important enriched pathways, functions and toxic functions in miRNA/mRNA interactions are shown; green and red colours indicate down- or up-regulation of miRNA or mRNA, respectively. Complete lists of enriched categories can be found in Table S9.
(XLS)

Tables S8 **Ingenuity Pathway Analysis (IPA) of enriched categories in transcriptome.** Statistical significance and complete list of involved molecules in each enriched categories are shown.
(XLS)

Tables S9 **Ingenuity Pathway Analysis (IPA) of enriched categories in miRNAs targets analysis.** Statistical significance and complete list of involved molecules in each enriched categories are shown.
(XLS)

Table S10 **Comparison of miRNAs dysregulations with previous studies.** The modulation of dysregulated miRNAs in DM2 patients were compared to previously published data on miRNAs dysregulation in muscular diseases. Green and red colours indicate down- or up-regulation of miRNAs, respectively.
(XLS)
