# Supplementary figures and images for: Deregulated MicroRNAs in Myotonic Dystrophy Type 2
Source: PLoS One. 2012 Jun 29;7(6):e39732. doi: 10.1371/journal.pone.0039732 (PMC3387258; doi:10.1371/journal.pone.0039732)

## Slide 1
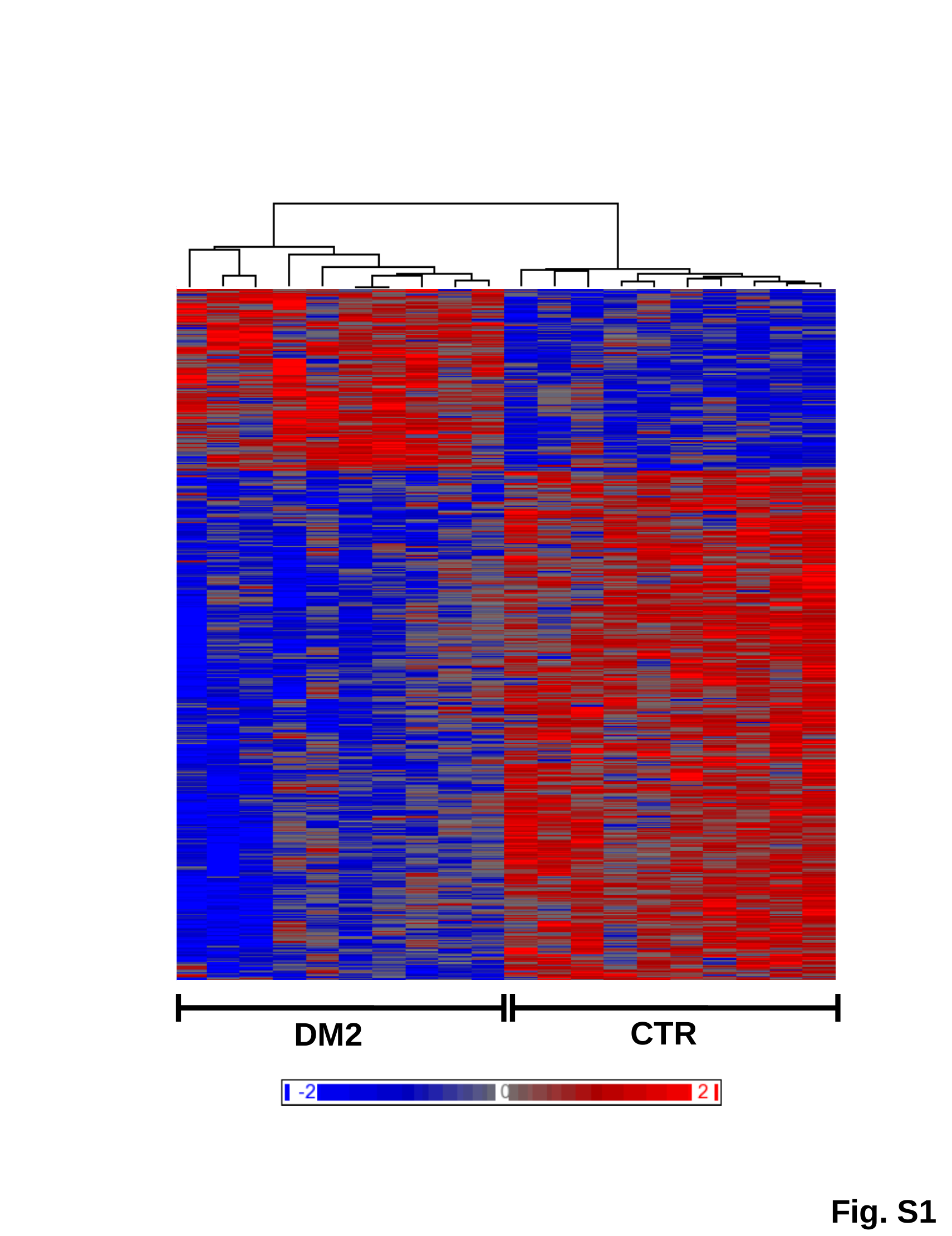

CTR
DM2
Fig. S1

Supplement: Figure S1 — Unsupervised hierarchical clustering of mRNA expression differentiating DM2 (n = 10) from controls (n = 10). Each row represents an mRNA, and each column represents an individual. A color code represents the relative intensity of the expression signal, with red indicating high expression and blue indicating low expression. (PPT) [file pone.0039732.s001.ppt]
